# Supplementary material for: Comparison of Kit-Based Metabolomics with Other Methodologies in a Large Cohort, towards Establishing Reference Values
Source: Metabolites. 2021 Sep 24;11(10):652. doi: 10.3390/metabo11100652 (PMC8538467; doi:10.3390/metabo11100652)
Supplement: Supplementary file 1 [file metabolites-11-00652-s001.zip › Supplemental Figures_R2.pdf]

Supplemental Materials

Addressing the accuracy of reference values in a large-scale cohort by kit-based metabolomics compared to other methodologies

Daisuke Saigusa [1,2*], Eiji Hishinuma [1,3], Naomi Matsukawa [1,2], Masatomo Takahashi [4], Jun Inoue [1,3], Shu Tadaka [1,5], Ikuko N Motoike [1,5], Atsushi Hozawa [6], Yoshihiro Izumi [4,7], Takeshi Bamba [4,7], Kengo Kinoshita [1,3,5], Kim Ekroos [8], Seizo Koshiba [1,3], and Masayuki Yamamoto [1,2,3]

[1]Department of Integrative Genomics, Tohoku University Tohoku Medical Megabank Organization, 2-1, Seiryo-machi, Aoba-ku, Sendai, 980-8573, Japan

[2]Medical Biochemistry, Tohoku University Graduate School of Medicine, 2-1, Seiryo-machi, Aoba-ku, Sendai, 980-8575, Japan

[3]Advanced Research Center for Innovations in Next-Generation Medicine, Tohoku University, 2-1 Seiryo-machi, Aoba-ku, Sendai, 980-8573, Japan

[4]Division of Metabolomics, Medical Institute of Bioregulation, Kyushu University, 3-1-1 Maidashi, Hi-gashi-ku, Fukuoka 812-8582, Japan

[5]Graduate School of Information Sciences, Tohoku University, 6-3-09, Aramaki Aza-Aoba, Aoba-ku, Sendai, 980-8579 Japan

[6]Department of Preventive Medicine and Epidemiology, Tohoku University Tohoku Medical Megabank Organization, 2-1, Seiryo-machi, Aoba-ku, Sendai, 980-8573, Japan

[7]Department of Systems Life Sciences, Graduate School of Systems Life Sciences, Kyushu University, 3-1-1 Maidashi, Higashi-ku, Fukuoka 812-8582, Japan

[8]Lipidomics Consulting Ltd., 02230 Espoo, Finland

*Correspondence: saigusa@tohoku.ac.jp; Tel.: +81-22-274-5925

Table of contents

P2: Figure S1, Figure S2

P3: Figure S3

P4-6: Figure S4

P7: Figure S5

P8: Figure S6

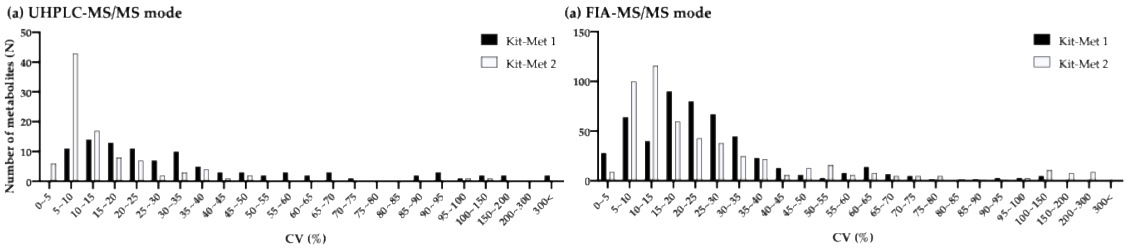

Figure S1. Frequency of detected metabolites along with the range of coefficient variation (CV, %) in gQC plasma at UHPLC-MS/MS mode (a, left panel), and FIA-MS/MS mode (b, right panel) by Kit-Met 1 (black bar) and Kit-Met 2 (white bar).

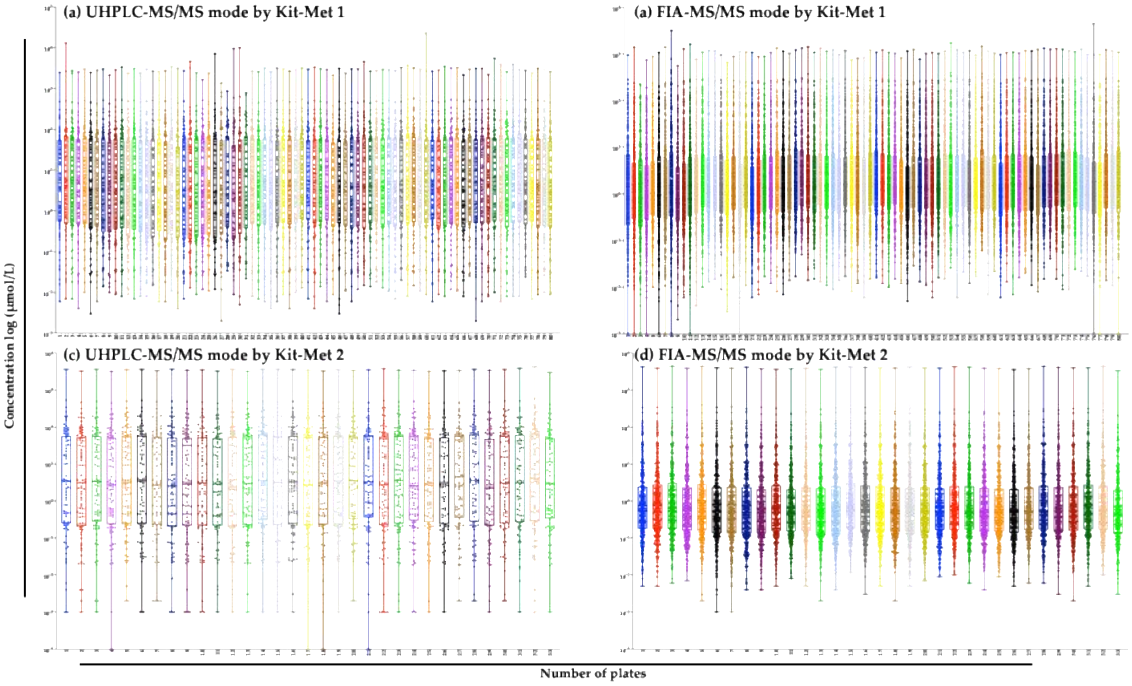

Figure S2. The median intensities of boxplot for detected metabolites in NIST plasma. The boxplots of 80 plates by Kit-Met 1 by UHPLC-MS/MS mode (a, upper left panel) and FIA-MS/MS mode (b, right panel), and the 33 plates by Kit-Met 2 by UHPLC-MS/MS mode (c, bottom left panel) and FIA-MS/MS mode (c, bottom right panel) were created and colored for each plate by GraphPad Prism 8.4.3.. Kit-Met 1 and Kit-Met 2 were performed by the Xevo® TQ-S and Xevo® TQ-XS MS system (Waters, Wilmslow, Manchester, UK), respectively. Both MS systems were conducted with the UHPLC system, which were consisted with dual pumps (ACQUITY UPLC H-Class, Waters) and an autosampler with a column compartment (ACQUITY UPLC I-Class, Waters).

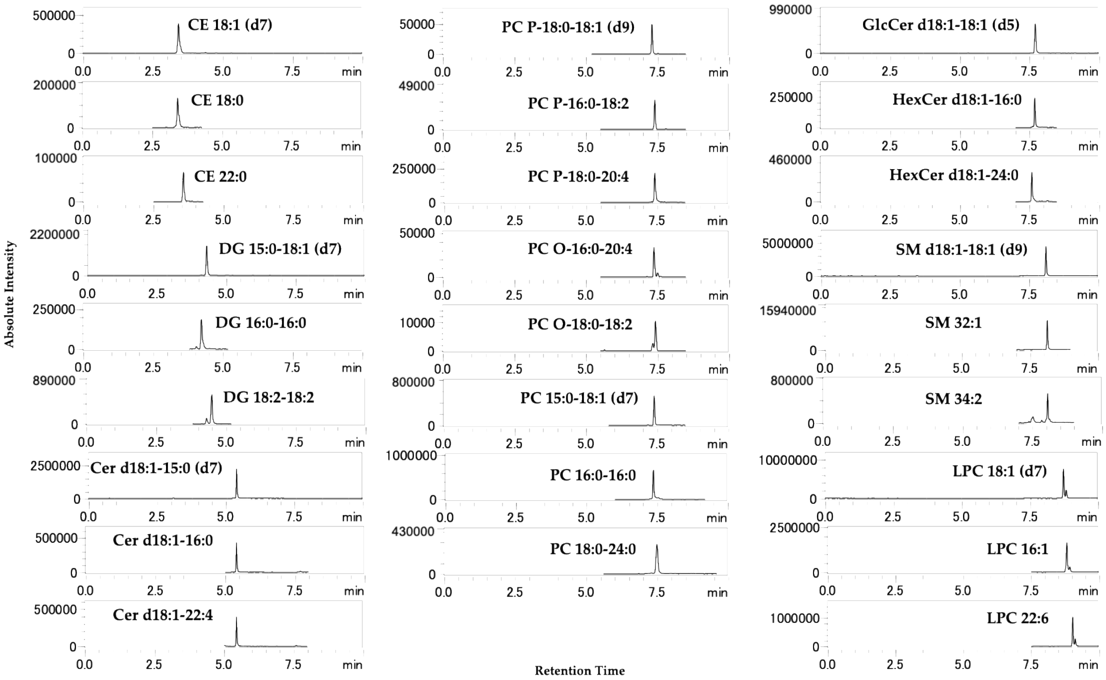

Figure S3. Multiple reaction monitoring chromatograms of representative lipid species detected in the NIST plasma samples by SFC-MS/MS.

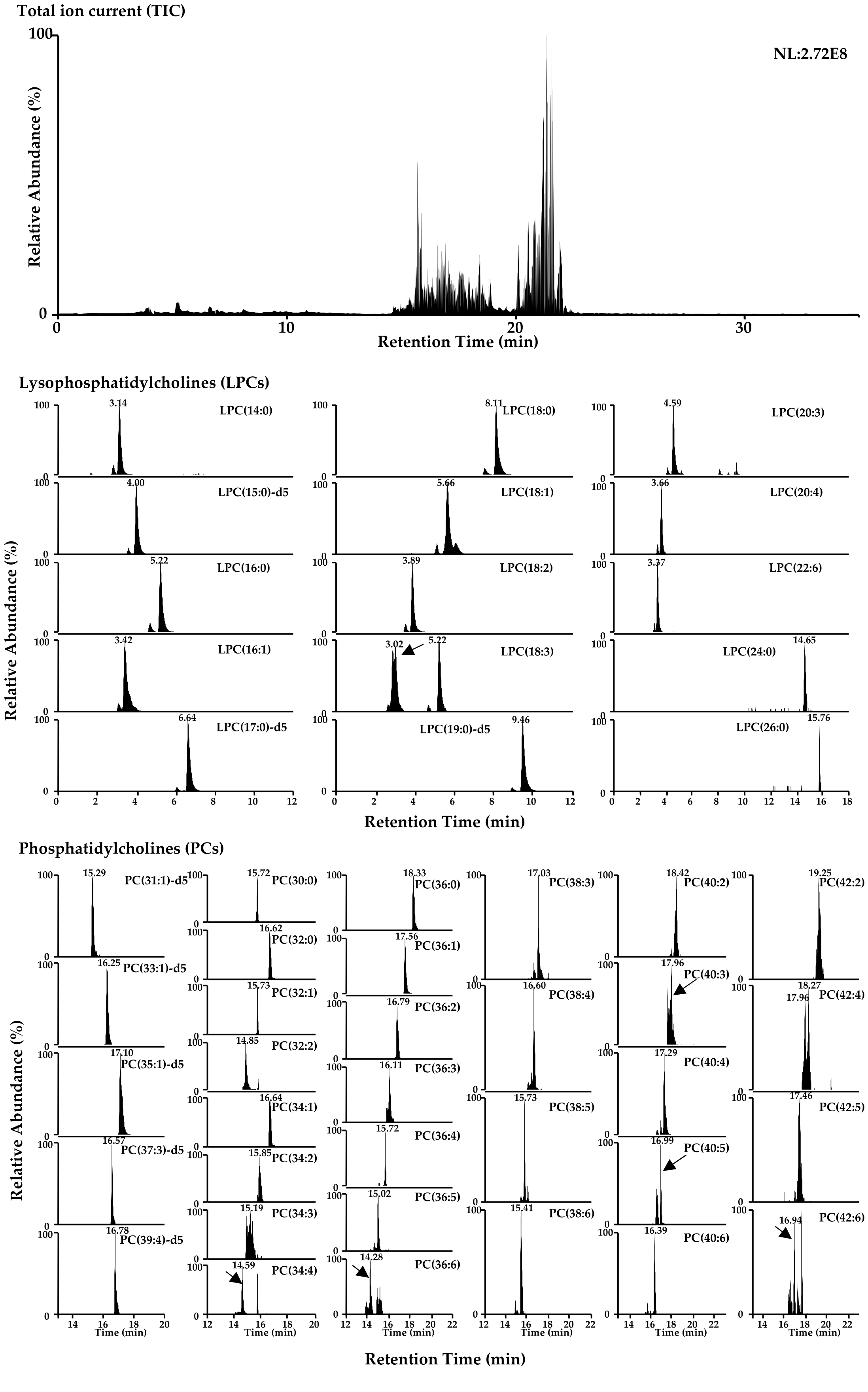
Total ion current (TIC)
NL:2.72E8
Relative Abundance (%)
Retention Time (min)
Lysophosphatidylcholines (LPCs)
LPC(14:0) 3.14
LPC(15:0)-d5 4.00
LPC(16:0) 5.22
LPC(16:1) 3.42
LPC(17:0)-d5 6.64
LPC(18:0) 8.11
LPC(18:1) 5.66
LPC(18:2) 3.89
LPC(18:3) 3.02 5.22
LPC(19:0)-d5 9.46
LPC(20:3) 4.59
LPC(20:4) 3.66
LPC(22:6) 3.37
LPC(24:0) 14.65
LPC(26:0) 15.76
Relative Abundance (%)
Retention Time (min)
Phosphatidylcholines (PCs)
PC(31:1)-d5 15.29
PC(33:1)-d5 16.25
PC(35:1)-d5 17.10
PC(37:3)-d5 16.57
PC(39:4)-d5 16.78
PC(30:0) 15.72
PC(32:0) 16.62
PC(32:1) 15.73
PC(32:2) 14.85
PC(34:1) 16.64
PC(34:2) 15.85
PC(34:3) 15.19
PC(34:4) 14.59
PC(36:0) 18.33
PC(36:1) 17.56
PC(36:2) 16.79
PC(36:3) 16.11
PC(36:4) 15.72
PC(36:5) 15.02
PC(36:6) 14.28
PC(38:3) 17.03
PC(38:4) 16.60
PC(38:5) 15.73
PC(38:6) 15.41
PC(40:2) 18.42
PC(40:3) 17.96
PC(40:4) 17.29
PC(40:5) 16.99
PC(40:6) 16.39
PC(42:2) 19.25
PC(42:4) 18.27 17.96
PC(42:5) 17.46
PC(42:6) 16.94
Time (min)
Relative Abundance (%)
Retention Time (min)

Ceramides (Cers)

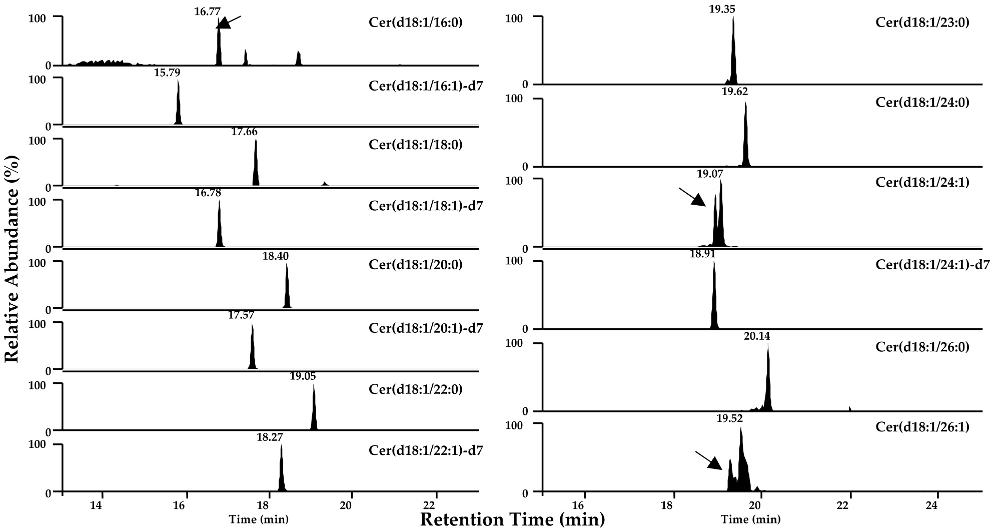

Sphingomyelins (SMs)

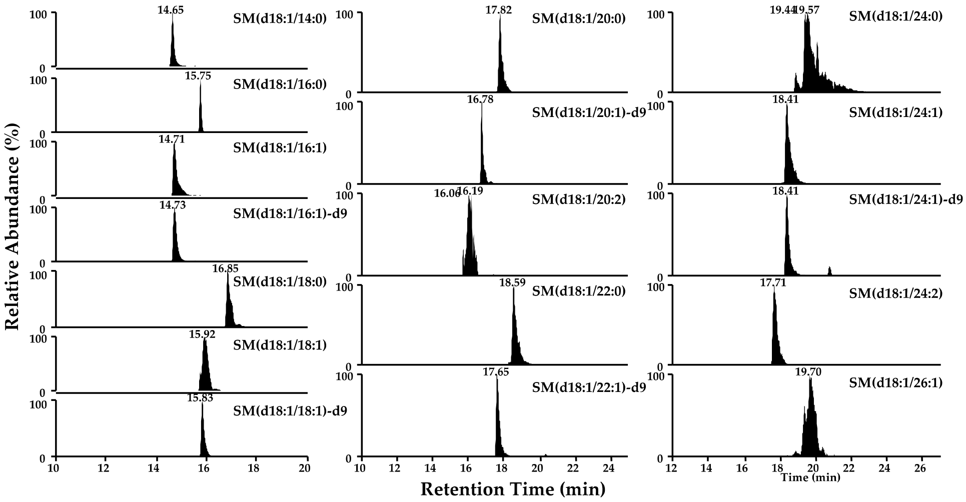

hexosylceramides (HexCers)

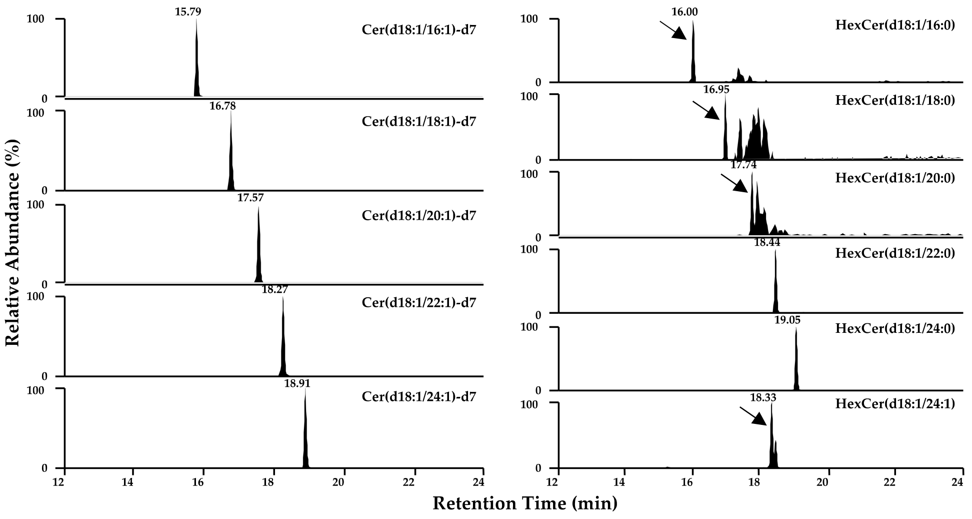

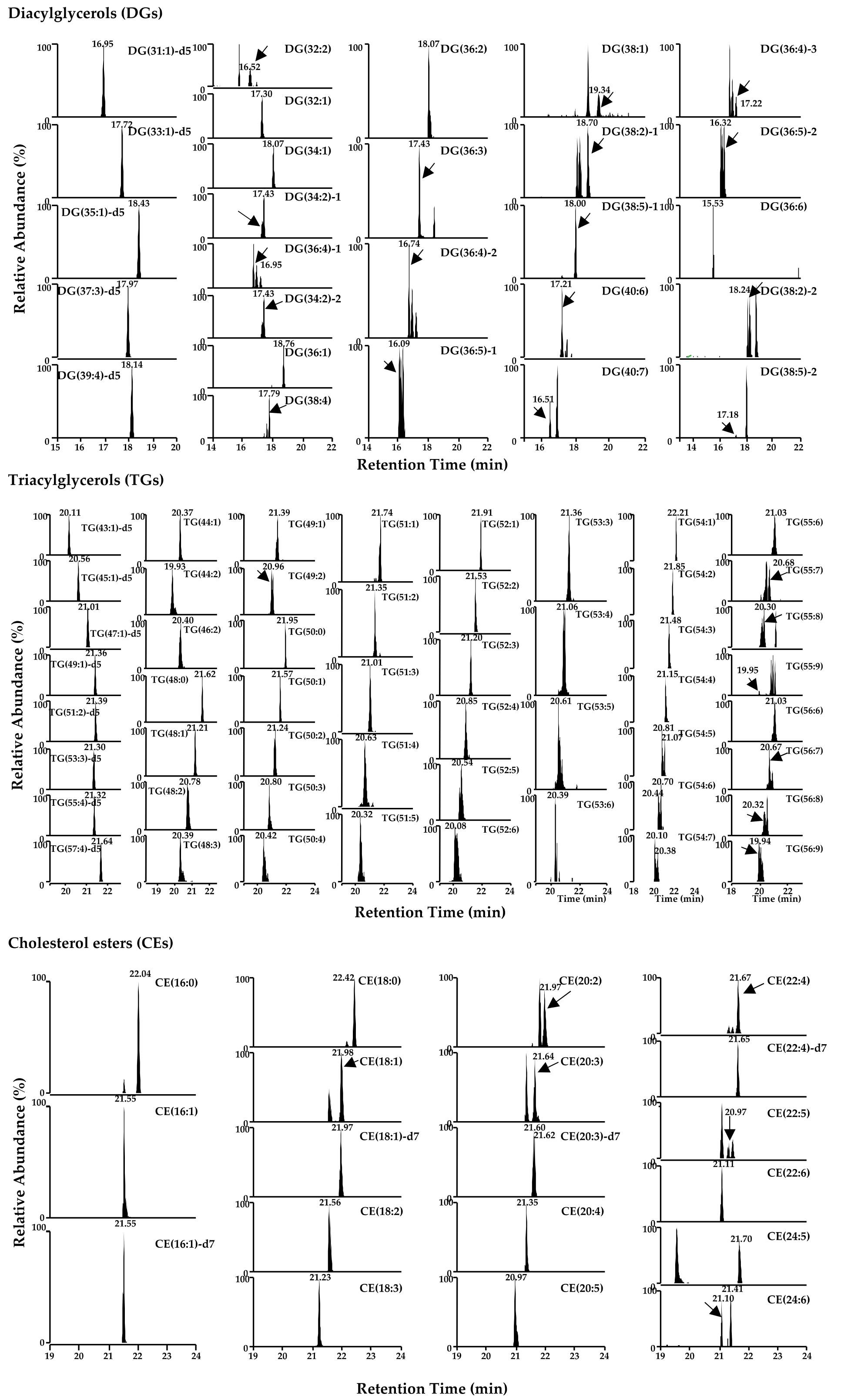

Figure S4. Total ion current (TIC) and mass chromatograms of 148 lipid species with ISs detected in the NIST plasma samples by UHPLC-FTMS at positive ion mode.

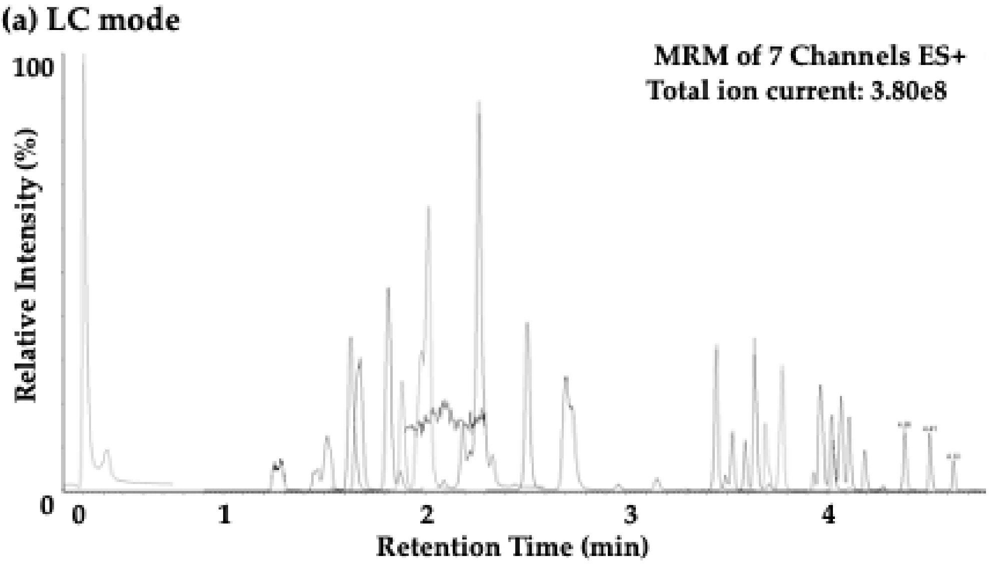

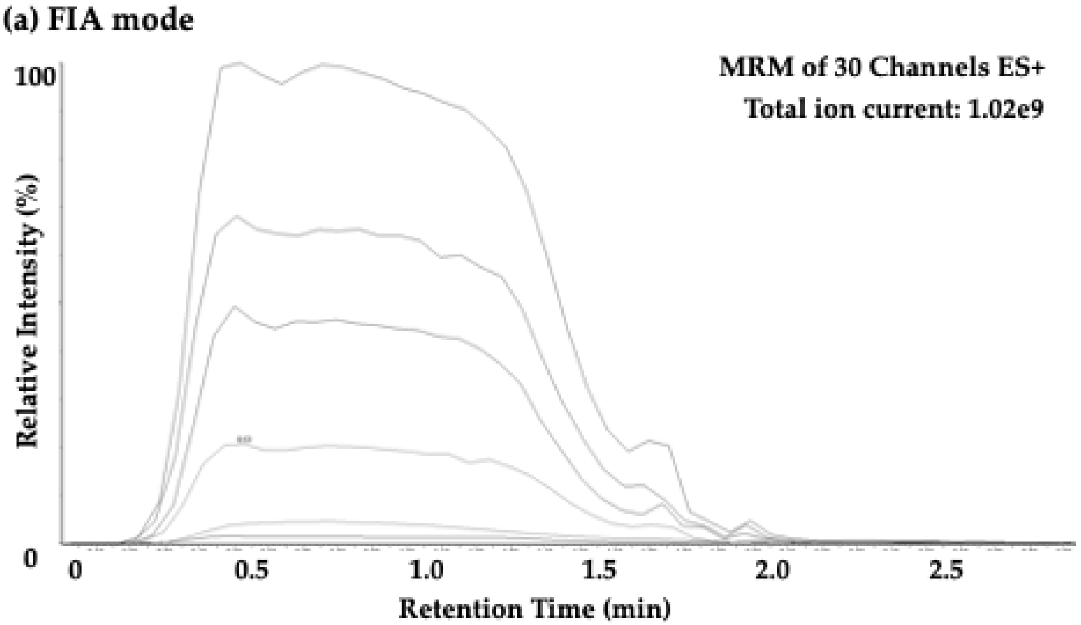

Figure S5. Examples for typical MRM chromatograms of NIST plasma sample analysis by Kit-Met 2 in UHPLC-MS/MS mode (a) and FIA-MS/MS mode (b).

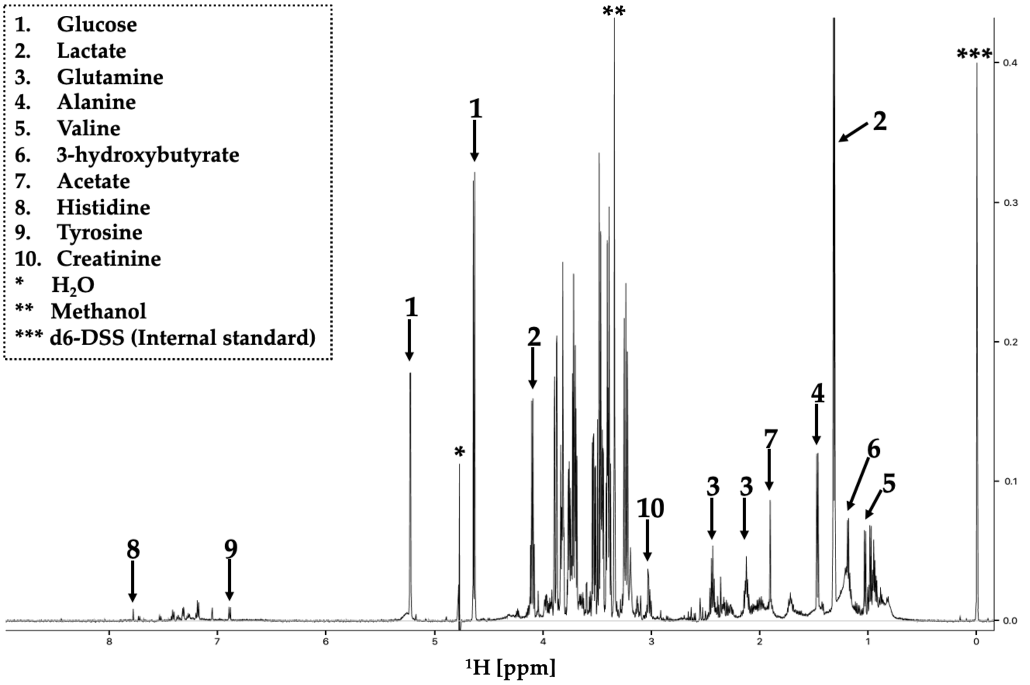

Figure S6. ^{1}H-NMR spectrum of detected metabolites in NIST plasma sample.
